# Notices of Some New Works

**Published:** 1839

**Authors:** 


### Periscope;

OR,

### CIRCUMSPECTIVE REVIEW.

"Ore trahit quodcunque potest, atque addit acervo."

### Notices of some New Works.

**Dr. Evory Kennedy on Hypertrophy and other Affections of the Os Uteri. Dublin, pp. 20.**

A ward set aside for the diseases of females in the Dublin Lying-in Hospital, has given our author the means of investigating a class of diseases which, for obvious reasons, cannot be extensively examined in this country at least. As the definition of a disease is of some importance, we shall give Dr. Kennedy's.

"What we wish to be understood by the term hypertrophy of the os uteri here made use of, is, the existence of a *partial* and extraordinary development of the uterus, in which, not only that particular portion, but even a particular tissue is engaged. The structure deposited appears to be the existing natural structure in excess; exhibiting little morbid or diseased character, other than that depending upon increased bulk and its consequences. Writers on uterine affections treat of general hypertrophy, or hypertrophy of the body and neck of the uterus, but this disease, as far at least as our observation goes, is rarely met with. We submit that the disease to which it is our object to call the attention of the profession is, on the contrary, one of very frequent occurrence."

The true nature of this hypertrophy is not, Dr. K. thinks, quite understood, some, as Boivin and Dugés, referring it to disease or chronic inflammation—others, as Cruveilhier, to exuberance of nutrition, with some degree of *mollescence*,—while Lisfranc considers it a morbid growth, with *hardening!* and fœtid discharge—an affection little analogous to that under consideration.

"With this general view of hypertrophy we must agree, not esteeming it at all imperative that any symptoms indicative of serious uterine disease should accompany it; we have at this moment in the ward for diseases of females, a case of general hypertrophy of the uterus, in which the only symptoms, in addition to the increase of bulk, are retroflexion of this organ, with slight sense of weight and occasional slight obtuse pain in the uterine region."

This hypertrophy may be either partial or general—the part most frequently affected, however, being one or other, or both of the lips of the os uteri. The disease has been mistaken for polypus, and the attempts to remove it have, of course, been fatal.

"The detecting, by a carefully conducted examination per vaginam, and if necessary by the rectum, the elongated or outstretched neck of the uterus, whilst the fundus of the organ is perceptible, of its natural size, and in its usual position in the pelvis, will sufficiently establish the diagnosis."

The affection under consideration, however, is one of the os uteri itself, though sometimes complicated with elongation of the cervix. It appears to consist of an erratic development of the intimate fibro-cellular structure of the part, owing either to an inordinate action of the secernant vessels—or diminution of action in the absorbents—"no altered or diseased action appearing to exist, beyond the loss of balance in the vessels specified." The new texture when cut into, exhibits the same characters as natural uterine structure.

"The aperture of the os uteri, in the majority of these cases, is found in its ordinary position, bearing the same relation to the roof of the vagina as in the natural state of the parts; the development of the new growth projecting below the opening. We shall best explain the nature and varieties of the affection by giving briefly a few of the cases presented to our notice.

The case of most frequent occurrence is that in which the anterior lip of the uterus takes on this excess of development. A patient with an affection of this kind came into Hospital January 2nd, 1838, æt. thirty-two, who had borne two children; menstruation regular; had for the last eight years been subject to occasional leucorrhœa, and latterly felt a tendency to the womb, as she says, descending, which was increased by laborious occupation. On examining with the speculum, the anterior lip was found protruding, and elongated to the extent of an inch and a half. It is free from pain, and gives the idea to the touch, of consisting of the natural texture of the uterus, being neither harder nor softer: the posterior lip is of the ordinary size, the vagino-uterine aperture small, found at the base of, and posterior to, the elongated tumor.

This case was treated for a fortnight with repeated leeching to the hypertrophied part, and alteratives. The inner surface of the tumour, which was slightly granular, was touched with nitrate of silver lotion.

She left the hospital on the 14th, reported as having the anterior lip considerably diminished in size, free from discharge, and in other respects improved.

In some instances, but more rarely, the posterior lip takes on this inordinate growth, and when this does occur, it is seldom to so great an extent. A case of this kind was under my care some months since, in which there was, as in the former case, some tendency to prolapse. The patient sometimes suffered pain in the region of the rectum, more especially when the bowels were constipated, or on making much effort at stool. The anterior lip was healthy, and the posterior projected upwards of an inch into the vagina, but was rounded and obtuse in its shape. This case was treated by leeching, alteratives, posture and otherwise, as for prolapsus, and with much benefit.

It occasionally happens that both the anterior and posterior lip become hypertrophied, and this more especially in women who have borne several children. In such, the fissured or divided state of the os, that so frequently results from labour, generally continues. The anterior and posterior lips presenting separate or distinct tumours. Such a case was under treatment, accompanied with complete procedentia of the uterus. In this patient the os uteri protruding beyond the external parts, exhibited not a bad representation of a bird with its beak separated. She was treated for prolapsus by cauterizing the vagina, a plan that I have been for some time successfully adopting, and with benefit to the procedentia, but was lost sight of before anything was done for the hypertrophy.

A more remarkable case, in which the os is fissured into three divisions, the intermediate substance becoming hypertrophied at three points, was accidentally discovered in a patient who came lately into hospital for the treatment of a vesico-vaginal fistula; both the fistula and the hypertrophy appeared to have been the result of a tedious labour in the country, where she was allowed to remain too long without assistance. The tripartite hypertrophied os is represented projecting into the vagina, but without any displacement of the uterus, or other symptom indicative of its existence. The outer surface of these three tumours is of the natural texture of the vagina, whilst the inner is rough, of a reddish tint, with vesicles interspersed, for which she has been touched with oxymel æruginis. It may be here remarked, that the internal surface of the hypertrophied portion generally exhibits more of the furrowed, irregular character of the intra-uterine mucous membrane, whilst the outer is generally polished and smooth; the internal and most dependent portions are not unfrequently vascular, or, as in the preceding case, present slight vesicles, or granular growths may exist upon their surface."

In most cases the hypertrophied portion is covered with mucous membrane, the continuation of that of the uterus or vagina. One case, however, is related as an exception.

"From the preceding cases it will be seen that the symptoms usually attending this growth, are a sense of fulness and weight in the upper part of the vagina, which may be accompanied with heat and throbbing in this region. As the disease progresses, the patient experiences a sensation as if a foreign body hung down into the vaginal passage; this is occasionally attended by a bearing-down sensation: and in cases of long-standing (particularly if injudiciously managed) may become converted into absolute prolapsus, or even procedentia of the organ. Now the practitioner may, for the first time, be aware of the existence of this preternatural growth of the os, when the error is very generally committed, of looking upon this development as the effect not the cause of the prolapsus. The preternatural growth, as well from its weight and mechanical bulk, as by the irritation it produces, acts as a foreign substance attached to the uterus. From its pressure upon the bladder, in hypertrophy of the anterior lip, interference is sometimes observed to occur with the functions of this organ: thus frequent micturition, and sometimes difficulty or extraordinary effort in discharging the contents of the bladder, occur; in other cases, particularly where the posterior lip is engaged, the functions of the rectum are interfered with, when costiveness or pain at stool occur.

On examination with the speculum, the lip of the uterus engaged is in some cases found scarcely at all altered in the appearance of its texture beyond the increased bulk; in other cases the hypertrophied portion appears red and vascular, or this vascularity may be confined to a part, generally the most dependent portion of it. It is also sometimes combined with granular disease of the os, although the menstruous secretion is seldom much interfered with. Hæmorrhagic discharges occasionally occur in this disease; and leucorrhœa is not an unfrequent accompaniment, more especially where much displacement of the uterus occurs. It is also not unfrequently productive of pain and inconvenience *in coitu*.

The throbbing, fulness, and sense of weight, may be increased about the setting in of the periodic discharge, indicating a congestive state of the part; if the speculum be now used, the hypertrophied portion may exhibit a more deep red or purple hue; from this train of symptoms the individual gets relief after the menstruous secretion has passed over. (Thus, no doubt, it has been treated as dysmennorrhœa in some cases.) The symptoms accompanying this affection at times partake more the character of irritable uterus, a disease so admirably elucidated by the late Dr. Gooch, at others, they appear to assume all those of inflammation in the tissue engaged, and sometimes they exhibit, as was the case in one of the patients mentioned, a mixed character, assuming symptoms both of irritation and inflammation, and combined with them the hypertrophied state of the os."

Where congestion or inflammatory action exists, repeated local depletions are necessary, with the warm hip-bath, and mild saline aperients. To these means mercurial alteratives are to be added. Our author recommends the local application of solution of nitrate of silver, or even the nitrate in substance. The recumbent posture is necessary. Dr. Hull's utero-abdominal bandage has been used with advantage by Dr. K. Pessaries should be avoided during the local treatment. The ring pessaries are the best, if this plan fails, and the prolapsed part induces serious inconvenience. This complaint is not *necessarily* accompanied by any of the symptoms above enumerated, as it may and does continue for years, without giving any indication of its existence, till the tendency to prolapsus attracts attention.

"The disease with which this affection is most likely to be confounded is polypus. Like polypus, the great extent of its surface is unattached, and its connexion with the uterus is at its upper or most distant part; its being connected externally with the uterus will distinguish it from intra-uterine polypus, whilst its not being pediculated will serve to distinguish it in general from polypus of the neck or os uteri. Its sensibility may assist in corroborating this point, but this symptom is not much to be depended upon. It has been justly explained by Dr. C. Johnson, (Dublin Hospital Reports,) that the absence of sensibility is but an equivocal test of polypus, whilst we must state that the presence of sensibility in the hypertrophied state of the os, is just as little to be relied upon."

Hypertrophy of the os uteri is much more frequently met with in the impregnated than in the unimpregnated state, for obvious reasons. It is a cause of tedious labour occasionally.

The practice to adopt in such cases is to press the protruding lip very gently upwards, and retain it there with the fingers during two or three pains. This is generally done with ease, as the tumor usually remains up while the head presses downwards. Many judicious observations and directions follow, for which we have not room here. Like all Dr. Kennedy's communications, this paper is practical, and therefore highly interesting.

